# Decoding the Pathophysiology of Non-alcoholic Fatty Liver Disease Progressing to Non-alcoholic Steatohepatitis: A Systematic Review

**DOI:** 10.7759/cureus.18201

**Published:** 2021-09-22

**Authors:** Sayma Yaqub, Patricia Ananias, Arpita Shah, Kanita Luenam, Arunima Mariya Jose, Joao Pedro Melo, Arifa Turkistani, Lubna Mohammed

**Affiliations:** 1 Internal Medicine, California Institute of Behavioral Neurosciences & Psychology, Fairfield, USA; 2 Public Health, University of Texas Health Science Center at Houston, Houston, USA; 3 Family Medicine, California Institute of Behavioral Neurosciences & Psychology, Fairfield, USA; 4 Internal Medicine, Sree Gokulam Medical College and Research Foundation, Trivandrum, IND; 5 Psychology, California Institute of Behavioral Neurosciences & Psychology, Fairfield, USA; 6 Pathology, California Institute of Behavioral Neurosciences & Psychology, Fairfield, USA; 7 Internal Medicine/Family Medicine, California Institute of Behavioral Neurosciences & Psychology, Fairfield, USA

**Keywords:** nonalcoholic fatty liver disease, nafld and obesity, bariatric surgery and nafld, steatosis nash, pro-inflammatory cytokines, leptin adiponectin, intestinal dysbiosis, toll-like receptors, nafld and natural killer cells

## Abstract

Non-alcoholic fatty liver disease (NAFLD) is the most common hepatic manifestation of metabolic syndromes, and its roots are strongly associated with obesity and insulin resistance. The excess fat induces inflammatory pathways by tissue irritation and progresses to non-alcoholic steatohepatitis (NASH), fibrosis, and has emerged as the most frequent cause of hepatocellular cancer (HCC). This systematic review was structured per the Preferred Reporting Items for Systematic Review and Meta-Analysis (PRISMA) guidelines. The evidence was obtained from 13 research articles published in PubMed, Google Scholar, and Science Direct databases, including cross-sectional, case-control, prospective cohort studies, meta-analysis, and systematic reviews. The inclusion/exclusion criteria of free articles, published in English involving humans of mid-age in the last five years were applied. This review highlights findings in 7781 individuals, including non-NAFLD, NAFLD, and NASH positive individuals based on anthropometric measurement, blood samples, FibroScan, flow cytometry, and liver biopsy. The results underscored that the onset of inflammation set on the background of NAFLD starts NASH; the understanding and control of inflammation will help us design definitive biomarkers and treatment modalities. The complex pathogenesis and comparatively slow advancement but high morbidity have led investigators to understand the nuts and bolts for early management and prevention. Lipotoxicity and dysbiosis stimulate the immune system to generate cytokines and chemokines and decline in adipokines. The role of proteinase3 (PR3) and antitrypsin (ATT) ratio and biliverdin reductase (BVR) compel the exploration for non-invasive tests for definitive therapy.

## Introduction and background

Non-alcoholic fatty liver disease (NAFLD) is defined as the deposition of greater than 5% fat in the hepatocytes by histology in non-alcoholics [1]. The sedimented triglycerides provoke inflammation and oxidative stress creating a spectrum ranging from non-alcoholic steatohepatitis (NASH) with simple microvesicular steatosis advancing to cirrhosis and hepatocellular carcinoma (HCC). For general populations, the prevalence rate of NAFLD is estimated to be approximately 20-30% in Western countries and 5-18% in Asia [2], making it the most common cause of chronic liver disease worldwide. The increasing healthcare consumption due to NAFLD and NASH has burdened the economy with 16.5 million cases in the United States (US) [3], and HCC associated with NAFLD has now risen to the incidence rate of 9% per annum [4]. NAFLD usually present as a component of metabolic syndrome (MetS). It is strongly associated with obesity due to the common insulin resistance mechanism causing increased deposition of free fatty acids. It is estimated that about 20-30% of adults in economically developed countries have excess fat accumulation in the liver; 50% among people with diabetes and about 80% in the obese and morbidly obese categories [5]. The risk of fatty liver was approximately 4.1 to 14-fold higher in people with high BMI [6]. However, although high BMI is the major risk factor, NAFLD is also diagnosed in individuals with normal BMI and categorized as lean NAFLD. 

The pathogenesis of NAFLD is a multifactorial and multistep process, involving the combination and interaction of genetic, demographic, clinical, and environmental factors, with up to 27% of heritability identified [7]. Many studies have explained that NAFLD in conjunction with obesity follows the classic “multiple hit” theory of NAFLD pathophysiology [8]. At the outset, the accumulated triglycerides induce cell injury secondary to oxidative stress, modified metabolism, protein misfolding, mitochondrial damage, and endoplasmic reticulum stress response creating a persistent chronic systemic inflammation state [8,9]. The continuous high-fat diet and energy imbalance results in adipose tissue hyperplasia and hypertrophy with altered hormonal and enzymatic activity directing body hemostasis towards immune and inflammatory overactivity [10].

Evolving facts point towards the translocation of swarming intestinal bacteria to hepatocytes through the common portal circulation known as intestinal dysbiosis as the primary instigator in NAFLD [11], and has been primarily involved in the stimulation of Toll-like receptor (TLR), the pattern recognition receptors, especially when encountering the gram-positive bacteria cell surface components (lipopeptides, peptidoglycan, and lipoteichoic acid) [8], and macrophages induction [12]. This also simultaneously stimulates Kupffer cells in response to damaged intrahepatic structural components known as danger-associated molecular patterns (DAMPs) [12,13]. The surge in pro-inflammatory cytokines such as interleukins (IL), tumor necrosis factor (TNF)-α, monocyte chemoattractant protein (MCP)-1 and plasminogen activator inhibitor (PAI)-1 along with the decline in anti-inflammatory cytokines (IL-7, IL-10, and adiponectin) enhances the effect of lingering inflammation [8,9,12]. The evidence of chronic injury and resultant inflammation is supported by the presence of acute-phase reactant pentraxin and high sensitivity c-reactive protein (hs-CRP) emerging as an important marker of liver fibrosis and steatohepatitis further confirming the responsibility for continued irritation [14].

Currently, liver biopsy is the benchmark for diagnosis and weight reduction with lifestyle modifications form the foundation of the treatment. The evidence of dietary changes with caloric restriction has helped in reducing hepatic fat load, and physical activity including aerobic workout has enhanced hepatic metabolism by increasing fatty acid oxidation, reducing conciliators, and safeguarding against steatosis [5].

In this article, we will elucidate the inflammatory cascade leading NAFLD to NASH by the TLRs, cytokines, and chemokines, alterations in hepatokines and adiponectin characterizing the inflammatory aspect of NAFLD and leukocyte cell-derived chemotaxin2 (LECTS) and natural killer (NK) cells linked to insulin resistance and form the common morbific insult for type 2 diabetes (T2D) and NASH. Though much has been done to treat insulin resistance, we are still learning about the NAFLD-NASH spectrum. The recognition of these intricate processes may help us find new non-invasive and easily accessible biomarkers and medical treatment options like anti-inflammatory agents and antibodies for a largely impacted obese population.

## Review

The systematic review on NAFLD in the obese population was conducted and documented according to the Preferred Reporting Items for Systematic Review and Meta-Analysis (PRISMA) guidelines [15] using PubMed as a primary database, and Google Scholar and ScienceDirect as additional sources.

A thorough literature search was conducted between April 7, 2021, and July 12, 2021. The following inclusion principles were applied to recognize the studies analyzing the relationship between obesity and NAFLD: human studies with the age limit of 45 years, irrespective of gender, free access to English texts, and articles published in the last five years. The articles used to compile this compact review include traditional reviews, systematic analyses, meta-analyses, clinical trials, case-control studies, cross-sectional studies, and cohort studies.

The keywords as shown in Table 1 were first examined individually and then in combination to get comprehensive data and association between obesity and NAFLD. First, the terms "NAFLD," "fatty liver," "NASH" were entered separately and then in combination. The Medical Subject Headings (MeSH) keyword search presented in Table 2 was used as a next step. The linked keywords with emphasis on metabolism, pathophysiology, and prevention, using Boolean letters AND, OR refined the results from 2403 to 100 studies for PubMed, from 2750 to 520 articles for Google Scholar, and from 112 to five papers for ScienceDirect data source.

**Table 1:** Keyword search using PubMed, Google Scholar, and ScienceDirect. NAFLD: non-alcoholic fatty liver disease; NASH: non-alcoholic steatohepatitis

DATABASES	Keywords	Total	Inclusion/Exclusion
PubMed	NAFLD OR fatty liver AND obesity	17,499	562
Google Scholar	NAFLD NASH	18200	1490
Science Direct	NAFLD NASH	11753	203

**Table 2:** MeSH keywords search using PubMed, Google Scholar, and ScienceDirect. MeSH: Medical Subject Headings; NAFLD: non-alcoholic fatty liver disease; NASH: non-alcoholic steatohepatitis

Databases	Search Strategy	Search Result	Inclusion/Exclusion
PubMed	((("Non-alcoholic Fatty Liver Disease"[Mesh]) OR "Fatty Liver"[Mesh]) AND "Obesity"[Mesh]((("Non-alcoholic Fatty Liver Disease/metabolism"[Mesh] OR "Non-alcoholic Fatty Liver Disease/physiopathology"[Mesh] OR "Non-alcoholic Fatty Liver Disease/prevention and control"[Mesh] )) OR ( "Fatty Liver/metabolism"[Mesh] OR "Fatty Liver/physiopathology"[Mesh] OR "Fatty Liver/prevention and control"[Mesh] )) AND ( "Obesity/metabolism"[Mesh] OR "Obesity/physiopathology"[Mesh] OR "Obesity/prevention and control"[Mesh] )))	2403	100
Google Scholar	NAFLD AND NASH AND Physiopathology	2750	520
Science Direct	NAFLD AND NASH AND Obesity Physiopathology	112	5

Results

**Figure 1:**
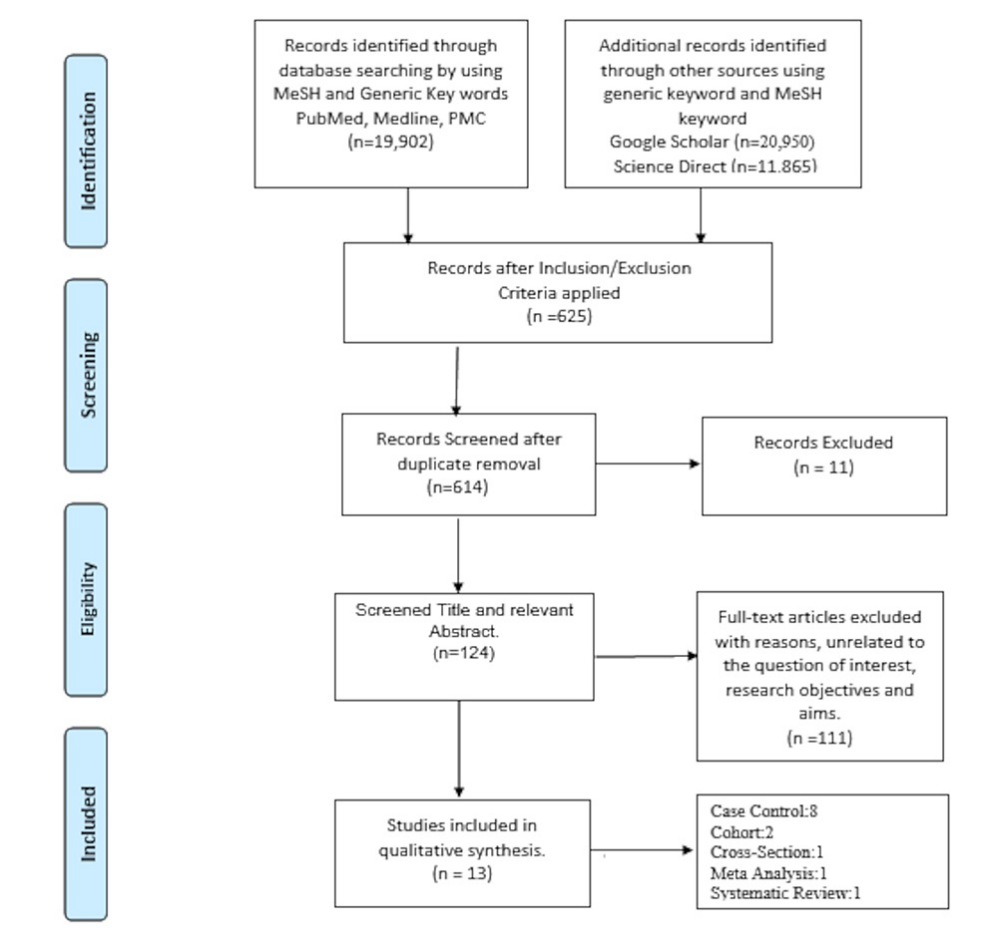
PRISMA flow diagram. PRISMA: Preferred Reporting Items for Systematic Review and Meta-Analysis n=Number of Articles

The result was compiled after screening the titles and abstracts relevant to research aims and objectives by two authors. Only moderate-to-high quality studies, focusing on NAFLD to NASH pathophysiology were included in the final analysis, and studies emphasizing solely the treatment, dietary influences, and relation to other diseases were excluded. The final study included a total of 13 papers as shown in the PRISMA flow diagram in Figure 1.

Table 3 shows the results of the studies included in this review. The selected studies have gathered information from 7781 participants: selected as healthy obese, obese with NAFLD, obese with NASH, and healthy controls. The results highlighted that activation of TLR-2, TLR-4, and TLR-6, in particular, activates cytokines and chemokines and lowers the level of adipokines. A new finding of elevated proteinase-3 (PR-3) and neutrophil elastase (NE) and lowered biliverdin reductase (BVR) ratio also illuminates the involved inflammatory pathway. The increased levels of plasma acute phase reactants like pentraxin and high sensitive c-reactive protein (hs-CRP) further confirmed this finding. The prominence of natural killer group 2D (NKG2D) receptors on CD56 NK cells needs further evaluation but also suggests the complete involvement of inflammatory pathways. These outcomes are linked with hepatic lobular inflammation and ballooning.

**Table 3:** Findings of the studies selected for the analysis. 1H-MRS: Proton magnetic resonance spectroscopy; AAT: aspartate aminotransferase; Apo: apolipoprotein; AST: aspartate aminotransferase; BVR-A: biliverdin reductase A; CCL3: chemokine ligand 3; CRC: colorectal carcinoma; ELISA: enzyme-linked immunosorbent assays; FACS: fluorescence-activated-cell-sorting; F: female; GGT: gamma glutamyl transferase; HsCRP: high sensitive C-reactive protein; H-MRS: proton magnetic resonance spectroscopy; (HOMA)2-IR: homeostatic model assessment method insulin resistance; iFAP: intestinal fatty acid binding protein; IL: interluekin; iFABP: intestinal-type fatty-acid binding protein; LAL: limulus amoebocyte lysate; LBPS: liposaccharide binding protein; LDL-C: low density lipoprotein cholesterol; LPS: lipopolysaccharide; M: male; MAIT: mucosa-associated T-cells; MDC: macrophage-derived chemokine; MCP: monocyte chemotactic protein; MIP-1α: macrophage inflammatory protein 1 alpha; MO: morbid obesity; mRNA: messenger ribonucleic acid; N: number; N/A: not applicable; NAFLD: non-alcoholic fatty liver disease; NASH: non-alcoholic steatohepatitis; NFS: NAFLD fibrosis scores; NE: neutrophil elastase; NK: natural killer cells; NKG2D: natural killer group 2D; NW: normal weight; PCR-RFLP: polymerase chain reaction-restriction fragment length polymorphism, PR3: proteinase-3, pDCs: plasmacytoid dendritic cells; PMBC: peripheral blood mononuclear cell; PTX3: pentraxin 3; RT-PCR: reverse transcriptase-polymerase chain reaction; RFLP: restriction fragment length polymorphism; sCD14: soluble form of cluster of differentiation 14; T2DM: type 2 dibetes mellitus; T-ARMS-PCR: tetra-amplification refractory mutation system-polymerase chain reaction; TC: total cholesterol; TG: triglycerides; TLR: toll-like receptors; TNF-α: tumor necrosis factor-α; tPAI-1: tissue plasminogen activator inhibitor-1; VAT: visceral adipose tissue

Author/Year of Publication	Type of Study	Characteristics	Method	Outcome
1. Auguet et al., 2016 [8]	Case-Control	N=111; Normal Weight=29; Morbid Obese=82	ELISA for cytokine and TLRs level measurement. RT-PCR hepatic TLR expression.	High IL-1β, IL-8, IL-10, TNF-α, tPAI-1, and MCP-1 levels were present in women with MO, and circulating adiponectin was low when compared to NW women. IL-8 was drastically high in women with MO with NAFLD, NASH group, and histologically correlated with hepatic lobular inflammation and ballooning
2. Ceccarelli et al., 2020 [10]	Meta-Analysis	N=38	PCR of omental biopsy samples in bariatric curgery candidates for BVR-A mRNA levels.	Lower BVR-A expression was associated with adipocyte hypertrophy, VAT dysfunction, high IL-8, and Caspase 3 expression, and NAFLD and high GGT
3. du Plessis et al., 2016 [11]	Prospective Cohort	N=111; Exposed=91; Controls=10	Plasma Biomarkers Plasma Endotoxin (LPS) using the LAL assay. ELISA for LBPS, Scd14, iFABP, TLR2,4 and Cytokine measurement (IL10, IL1β, IL6, IL8, TNF-α,) and chemokine (MCP1 and MCP4), MDC, MIP-1α/CCL3 levels.	Plasma LPS is a marker of metabolic syndrome. The Plasma sCD14 and iFABP are not elevated across the histological subgroups of NAFLD. The plasma TLR receptors were high among the obese population, but cytokine and chemokines were significantly high in liver inflammation.
4. Trojak et al., 2019 [14]	Case-Control	N=116; Case (Diabetes+ NAFLD) =79; Controls (Diabetes)=37	Fasting serum lipids by Roche re‑agents. Cytokeratin 18 fragment – a marker of NAFLD – PTX3, apo C3, B100, and B48 were determined by ELISA. Liver ultrasound for NAFLD.	The PTX3 levels increase with an expansion in serum lipids and apolipoproteins. Cases have a strong correlation with TC, LDL-C, TG, apoC3, apoB. Controls showed a definite association with TC, TG, waist circumference, and BMI.
5. Bekaert et al., 2016 [16]	Case-Control	N=108; Cases:90; Controls=18; M:F= 75:33	ELISA test to measure serum adipokines. Gene expression analysis for VAT biopsy samples obtained from the gastrosplenic and gastrocolic ligaments. MCP-1, omentin, and chemerin were calculated by real-time PCR. Histological analysis of lateral liver edge biopsy samples.	The serum adiponectin levels were lowest in NAFLD patients. The serum MCP-1 levels were lowest in the obese without NAFLD. VAT expression of Omentin, SFRP4 with low chemerin expression in NAFLD with no difference in MCP-1.
6.Baltieri et al., 2018 [17]	Cross-sectional	N=19	Western Blot analysis at a 540 nm wavelength for the plasma levels of adiponectin, leptin, resistin, IL-6, IL-8, TNF-α, and CRP, in fasting state. Liver biopsies to assess NAFLD and categorized as steatosis, fibrosis, and steatohepatitis.	Mild steatosis was found in all participants. Inverse relationship was found between steatosis and fibrosis, and the levels of adiponectin.
7. Divella et al., 2019 [18]	Case-Control	N=215; Case (colorectal cancer)=165; Case (healthy=50)	Serum samples for ADIPQQ, leptin, and TNF-α were tested by a sandwich ELISA assay, genotyping of DNA extracted from blood. ADIPOQ RS266729 polymorphism detected by T-ARMS-PCR. TNF-α genotyping by PCR-RFLP.	Low adiponectin, high leptin, and TNF-α levels were associated with obesity, NASH, and play an important role in CRC-hepatic metastasis.
8. Mirea et al., 2019 [19]	Case-Control	Four cohorts were used. N=918; Cohort 1 (obese with liver steatosis) =271; Cohort 2 (biopsy-proven NAFLD) =41; Cohort 3 (obese, diabetes) =401; Controls (lean, healthy)=205	Estimation of hepatic fat by localized 1H-MRS. ELISA for plasma PR3, NE, AAT, and hsCRP estimation liver lysate for hepatic PR3, NE.	Plasma PR3 was high among patients with hepatic steatosis.PR3 and NE levels are associated with NASH/liver fibrosis. PR3 and NE upregulated in T2DM obese cohort.
9. Stiglund et al., 2019 [20]	Case-Control	N=67; Cohort 1 (biopsy-proven NAFLD)=26; Cohort 2 (gastric bypass surgery candidates)=26; Controls (healthy, lean)=15	Peripheral blood NK cells isolation by density gradient centrifugation. (Flow cytometry) liver biopsy samples were evaluated by flow cytometry staining, microscopy, checked for functionality.	A decline in MAIT, pDCs, in NASH. Plasma NK cells were unchanged in NAFLD/NASH. Plasma CD56 (bright and dim) shows upregulated surface NKG2D receptors. Hepatic and adipocyte resident NK cells were intact.
10. Arias-Loste et al., 2020 [21]	Prospective Cohort study	N=40; NASH=17; Non-NASH=17; M:F=15:25	FACS Analysis of PMBC for TLR2/TLR6 Expression. Immunohistochemistry of liver biopsy samples for TLR2/TLR6 expression.	No difference in TLR2 and TLR6 of PMBC between healthy and morbidly obese. High TLR6 in NASH PMBC. Higher Expression of TLR6 IN NAFLD and NASH patients. e IL-1β, TNF-α, and IL-6 are significantly high in NAFLD and NASH.
11. De Nooijer et al., 2019 [22]	Systematic Review Article	N/A	The difference between histology of pediatric and adult NASH along with data compilation on effects of diet, lifestyle choices, genetics, and androgen hormonal changes	The detrimental effects of high carbohydrates particularly fructose and fatty diet, genetic tendency, protective estrogens, and negative androgens influence NAFLD.
12. Diedrich et al., 2020 [23]	Case-Control	N=71; NAFLD PBMC n=27; NAFLD liver n=15; Control PBMC n=26; Control liver n=3	The 16-color flow cytometry to evaluate the phenotype of 23 immune sub-cell types and FibroScan to determine hepatic fibrosis.	The inverse relationship was noticed between hepatic fibrosis and the quantity of circulating NKG2D iNK cells. In NAFLD cases total CD3+, CD8+ T cells, CD56dim NK cells, and MAIT cells were low with high levels of CD4+ T cells and Th2 cells. The intrahepatic total T-cells, total CD8+ T-cells, Vd2+γδ T-cells, and CD56 bright NK cells were low with high Vδ2-γδ T-cells and CD56 dim NK cells when compared to controls.
13. Chou et al., 2021 [24]	Case-Control	N=5967; three groups: Betel nut current-chewers, ex-chewers, and non-chewers.	The AST/platelet ratio index and NFS were calculated for evaluation of liver fibrosis	Significant risk of fibrosis in NAFLD patients with current or ex-betel nut chewing but no effects of betel nut chewing in non-NAFLD individuals.

Discussion

In this review, we have compiled the information from 13 studies that highlight the activation of the Inflammatory pathway starting from TLRs, the imbalance between pro- and anti-inflammatory cytokines including adipokines in visceral adipose tissue (VAT), and the involvement of NK cells involved in the progression of NAFLD to NASH.

The Role of TLRs

TLRs are the component of innate immunity and may be present on the cell surface (TLR1, TLR2, TLR4, TLR5, and TL6) to detect the bacterial and microbial antigens or associated with the intracellular vesicles (TLR3, TLR5, TLR7, TLR9) and responsible for identifying the nucleic acid derived particles [5]. The latter are called pattern recognition receptors (PRP) and microbial surface antigens are called pattern-associated molecular patterns (PAMPS). Many animal studies suggested the strong occurrence of TLR2, TLR4, TLR5, and TLR9 in NASH [8], but in recent studies, the high concentration of TLR6 in peripheral monocytes, B-cells, and hepatocytes is the hallmark finding and emerging as the committed peripheral biomarker for obesity-associated NASH. The cycle begins when activated (TLR4, TLR6) signal downstream adaptor molecules, MyD88. The generation of proinflammatory cytokines and antimicrobial products is prompted by intermediary nuclear factor kappa-light-chain-enhancer of activated B-cells (NFκB) and mitogen-activated protein kinases (MAPK) [21]. The low TLRs in non-NASH obese liver biopsy samples as compared to NASH positive samples has instigated research in this direction to understand NASH pathology.

TLR2 has been proven to be greatly involved in NASH inflammation either by activating Kupffer cell inflammasome or forming heterodimers with TLR6 on recognizing gram-positive diacylated lipopeptides. The animal model with dysfunctional TLR2 did not develop expected insulin resistance and pro-inflammatory surge when diet-mediated NASH was induced [10]. The continued exposure to dysbiosis not just boosts NAFLD to NASH but the compositional change in microorganisms' surface molecules increases the delivery of new and robust TLRs to the liver, further enhancing the inflammatory soar.

TLR4 is expressed on hepatic parenchymal and non-parenchymal cells. The phagocytosis of saturated fatty acid and cholesterol particles, TLR4 is upregulated and promotes tissue injury, inflammation, fibrosis, and progression to HCC and the obese HCC population has shown evidence of TLR4 messenger RNA (mRNA) with interleukin 6 and interleukin 10 (IL6 and IL10) expression [22,25]. Arias-Loste et al. have demonstrated the high TLR6 cell surface expression in peripheral blood mononuclear cells (PBMC) and inflamed hepatocytes of NAFLD and NASH patients concurrently resulting in pro-inflammatory cytokines like e IL-1β, tumor necrosis factor-alpha (TNF-α), and IL-6 release in different quantities when TLR2 or TLR2/TLR6 were stimulated. It has been hypothesized that although the absence of IL-6 may exclude NASH in NAFLD, it does place these patients at the borderline and progress is imminent [21].

This role of TLRs is further confirmed by the action of newly developed anti-cytokine drugs like resatorvid (or TAK-242) which is a TLR4 inhibitor [25]. So far developed to treat septic shock, they could also be efficacious to control continued systemic inflammation seen in NASH development. The measurement of TLRs in blood as non-invasive biomarkers may help clinicians in apt diagnosis as well as to keep track of the disease progression. 

Pro-Inflammatory and Anti-Inflammatory Cytokines

The metabolic stress due to lipotoxicity, intestinal dysbiosis, and TLRs activation triggers the immune system. In response, the mobilized M1-type macrophages, monocytes, and hepatic stellate cells along with activated potentiation factors like kinases and transcription factors stimulate the proinflammatory cytokines and chemokines flow (IL-1β, IL-8, IL-10, TNF-α, tPAI-1, MCP-1). This disequilibrium also shows vanishing anti-inflammatory cytokines (IL-7, IL-10, and adiponectin) at the same time [7].

High leptin levels and insulin resistance are some of the initial pathways of metabolic syndrome in obesity and NAFLD. The high levels of leptin boost TNF-α and IL-6 release from hepatic stellate cells during early stages to perpetuate inflammation severity [4] and unwanted abundance of pro-angiogenic factors like vascular endothelial growth factor (VEGF) and hypoxia-induced factor-1α (HIF-1 α), increasing the progression to HCC [17]. IL-8 recruits and potentiate macrophages to induce inflammatory microenvironment by polynuclear chemotaxis and generation of cytokines, chemokines, and fibrogenic factors to extend the vicious cycle. The increased levels of IL-8 show a strong correlation with fibrosis, hepatocyte ballooning, and cirrhosis among individuals with NAFLD [8]. Jarrar et al. have demonstrated high IL-8 among individuals with NAFLD compared to healthy controls. [18]. The autocrine effects of IL-8 on its receptors in adipocytes further maintain inflammation progression [26]. The expression of tissue-like growth factor-β3 (TGF-β3) in NAFLD and tumor-like growth factor-β1 (TGF-β1) gene in NASH has shown distinction from the healthy counterparts [27].

The IL-1β is converted to bioactive form by caspase-1 of NLRP3 inflammasome protein complex but Mirea et al. have shown that the neutrophil serine proteases like NE and PR3 have also been used by liver neutrophils to activate it [19]. The study showed a disparity between NE, PR3, and their inhibitor antitrypsin (ATT) correlate with the severity of steatohepatitis when compared with the mild disease. The NE was higher among liver steatosis individuals and ATT was higher among lean healthy adults. The PR3 to ATT ratio was also high in the steatosis group [19]. These levels are linked optimistically with hs-CRP a chronic injury marker and it is worth reminding that hs-CRP serves as the marker of disease gravity when compared from steatohepatitis to NASH [19,27]. Pentraxin, an acute phase reactant resembling CRP was found to be raised in patients with hepatic fibrosis when compared to patients with NAFLD and non-NAFLD. It has a dual role in the inflammatory pathway. It counteracts IL-1, TNF- α, infectious agents induced inflammatory route but, on the other hand, it has strong connections with apolipoprotein (Apo)-C3, Apo-B100, which serves as a marker for atherogenic hepatic lipoproteins like very-low-density lipoprotein (VLDL) and sdLDL small density LDL (sdLDL) which are involved in direct systemic inflammation [14].

This data is in line with the findings of Zang et al. suggesting high sensitivity and specificity of NE/AAT ratio [28]. Since PR3 and NE are playing an important role in activating cytokines and propagating inflammation reactions, more data should be collected to learn them as disease activity markers and employing ATT as a therapeutic agent. The loss of equilibrium is simultaneously contributed by the diminished activity and synthesis of anti-inflammatory cytokines like IL-10, IL-7, IL-4 IL22, INF-gamma (IFN-γ), and adiponectin. Notably, IL-22 has an anti-inflammatory role by provoking hepatic repair but over time, under STAT3 overexpression, this induced IL-22 can turn on its proinflammatory and tumorigenic component [8]. During the last few years, BVR-A, an antioxidant enzyme of heme metabolism conduit emerged as a novel regulator of the insulin/IGF-1/PI3K/MAPK signaling cascade, and the lower levels of BVR-A in PBMC and adipocyte mRNA expression are associated with high IL-8 and caspase3 release. The proapoptotic state due to high CASP3/7 and BCL2 expression also joins the threads to inflammatory markers and insulin resistance seen in NASH as shown by Tinahones et al. [29].

In one of the unique case-control studies conducted by Chou et al., the relationship between betel nut chewing and the evolution of NAFLD to NASH and fibrosis was highlighted. They found out that ex and current betel nut consumers had high evolution to fibrosis among NAFLD patients as compared to non-chewers. The toxic metabolites of safrole and arecoline in betel nut tobacco not just self-induce reactive oxygen species but also prompt crossroad CYP2A6 and CYP2C9 of cytochrome p450 system creating an inflammatory torrent of TNF-α and NFκB. The study also expounded that aging, smoking, diabetes, male gender, and obesity were more likely to propagate fibrosis and NAFLD expansion to NASH [24].

By learning more about these inflammatory markers, non-invasive interventions can be devised for rapid testing of newly reported NAFLD and monitoring disease progression. The remarkable success in suppressing CCL24 chemokine by monoclonal antibodies in hepatic inflammation and fibrosis murine models is making our way towards treatment options.

Adipokines in VAT

Adipokines are a group of secretory substances with anti-inflammatory properties produced by adipocytes and play an influential role in hepatosteatosis progressing to fibrosis. The improvement in adipokines and NAFLD post-bariatric surgery is indirect evidence of its importance and positive effect in the process [17]. The key identified adipokines include adiponectin, leptin, resistin, omentin, chemerin, MCP-1, and secreted frizzled-related protein4 (SFRP4) [16].

The anti-inflammatory and anti-tumorigenic adiponectin is a polypeptide hormone and, along with omentin, not just increases insulin sensitivity but also shows cardioprotective effects [13]. The adiponectin has an antithetical link with the grade of hepatic steatosis, fibrosis, and NAFLD Activity Score (NAS score). There is a strong connection between insulin resistance and NASH. It is not just anti-inflammatory but also inhibits IL-6 and TNF-α, which as discussed are the pioneer provocateur [19,22]. IL-6 is very well known for insulin resistance. Divella et al. presented that obesity, insulin resistance, and altered adipocytokines (low adiponectin, high leptin, and TNF-α) not just increase the risk of colorectal cancer (CRC) but also lead to CRC hepatic metastasis due to NAFLD mediated liver inflammation [18].

Leptin has a twin role in NAFLD [4]. Primarily at the early stages of the disease, it plays a shielding role against hepatic steatosis [30], soon after enlargement of VAT, leptin resistance ensues, mostly due to suppression of cytokine3 (SCCS-3), which is involved in leptin inhibition when overexpressed, and insulin resistance. The stellate cells influenced by excessive leptin enter the positive loop of fibrosis thereby promoting proinflammatory and fibrinogenic adipocytokine [22]. This is further clarified by D’Incao et al. presenting a negative correlation between leptin and resistin with steatosis and fibrosis, respectively [30].

The other key player adipokine expressed in VAT, subcutaneous adipose tissue (SAT), and measured in the serum is chemerin. Chemerin induces chemotaxis and insulin resistance along with fibrogenic MCP-1. The elevated chemerin levels have been described frequently in patients with metabolic syndrome and have been associated with BMI, serum glucose, triglycerides (TG), high-density lipoprotein (HDL) cholesterol levels, and blood pressure [17].

The study by Bekaert et al. demonstrated low VAT chemerin expression and high serum chemerin in obese patients with diagnosed NAFLD as compared to controls but its association with hepatic histopathologic grading is still not clear [16]. Many studies have suggested detrimental chemerin visceral tissue expression leads to hepatic ballooning and steatosis irrespective of obesity, the homeostatic model assessment method insulin resistance (HOMA-IR), and type 2 diabetes mellitus. It has been suggested that since portal circulation is the major hepatic blood supply getting a major chunk of chemerin through the VAT, systemic chemerin levels can be elevated due to contribution from SAT. Other adipokines like omentin, SFRP, and MCP-1 and their contrasting high adipose tissue expression in NAFLD with low serum levels are still in the stages of investigation.

NK Cells

As part of the innate immune system, the NK cells originate from common lymphoid precursor cells in the bone marrow. Although NK cells account for only 2-18% of the lymphocytes in human peripheral blood, they compose up to 30% of the lymphocytes in the liver, consisting of half CD56bright and half CD56dim cells with unique phenotypes and functions [31]. NK cells have always been vital in hepatitis C- and D-induced cirrhosis and HCC and ensuing associated diversification of NK cells. On the other hand, NASH is also a slow but progressive inflammatory process, and the degree of fibrosis is of prognostic value. As discussed, cytokines and chemokines trigger the vicious circle and recruit immune cells including NK cells. The chemokines MCP-1, CCL5, and CCLX10 released by the steatotic liver are proven to corroborate NK and mononuclear cells. The murine models have shown the contribution of NK cells in hepatic steatosis by upregulated surface ligands as well cytotoxic NK cell influx by TNF-α induced apoptosis ligand (TRAIL) generating proinflammatory force [20].

The results from the study performed on the human circulating NK cells and the hepatic and adipose tissue-resident NK cells by Stiglund et al. suggested that there could be overexpression of circulating NK cell surface ligand (NKG2D) but no changes in influx or repertoire in native NK was noted in hepatic and adipose tissue. NK cells phenotype was even unaffected by the disease severity, degree of fibrosis, insulin resistance, or obesity among the cases and controls. But at the same time, the study presented the limitation that due to multivariate compartments within these tissues the exact function and analysis are difficult in human subjects [20]. In contrast, the results by Diedrich et al. also showed an inverse relationship between NK cell number and fibrosis. The lower quantity of NKG2D+ total and CD56dim NK cells as well as drastically decreased mean fluorescence intensity (MFI) of NKG2D on total NK cells and CD56dim NK cells in PBMC and hepatic compartment of NAFLD patients compared to healthy individuals was recorded. They concluded that NK cells play a regulatory role in fibrosis and steatosis progression and their low frequency in NAFLD and NASH as compared to healthy controls suggest protective function [23].

**Figure 2:**
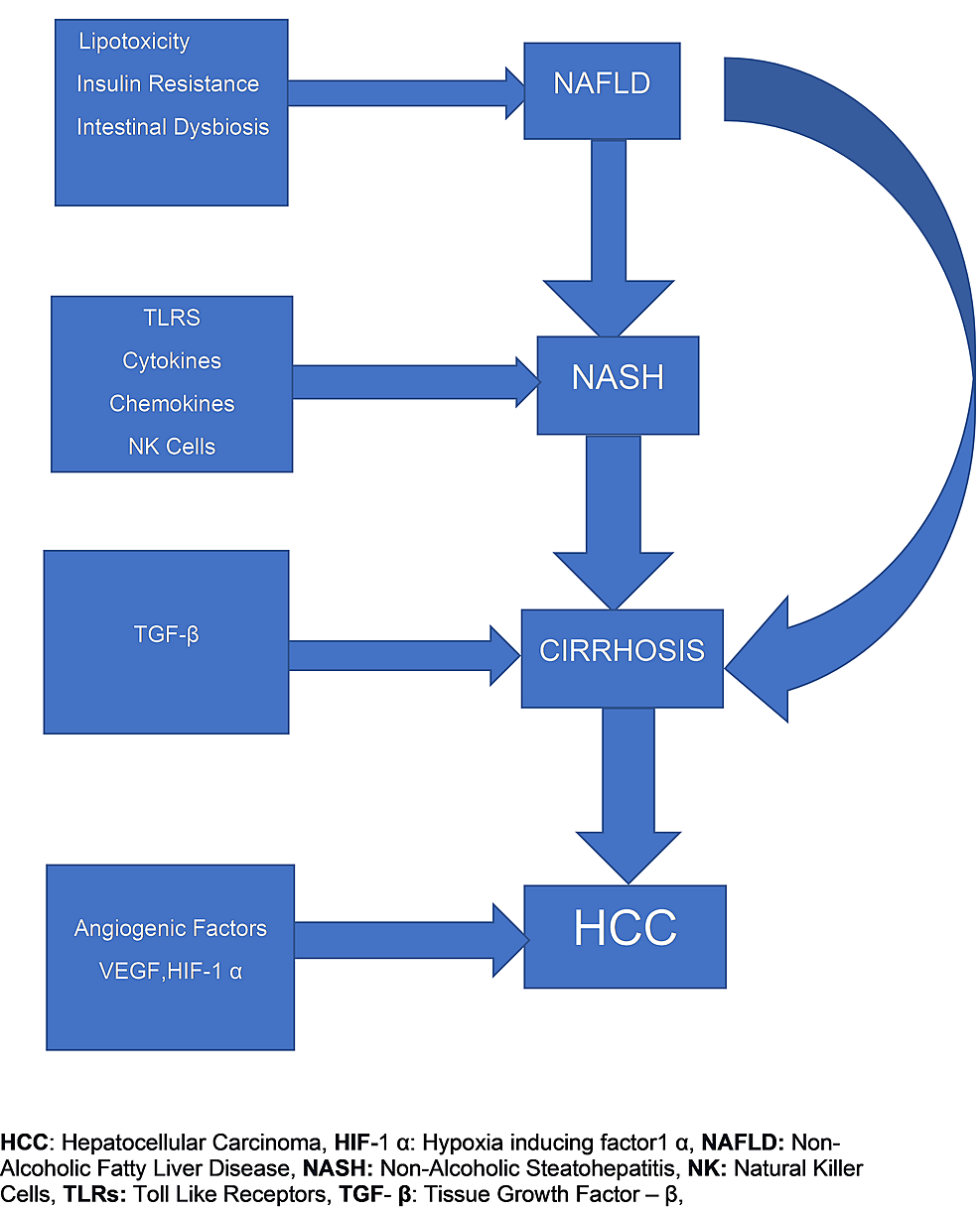
The pathophysiologic progression of NAFLD to NASH, fibrosis, and HCC. VEGF: vascular endothelial growth factor

One of the studies on the human adipose tissue NK cell population by O'Rourke et al. conducted in 2013 provided an initial insight into the NK cell activity in the adipose tissue. It showed that the activated NK cells in obese populations express high occurrence of cell surface receptors as NKG2D, CD158, CD16dim, NKp46 and CD27+ were highest in visceral adipose tissue samples in comparison to peripheral blood NK cells of obese and no difference was found in peripheral blood NK cells and VAT of the lean comparison group [32]. The important modulator of adipose tissue macrophages and insulin resistance is IFN-γ, which is secreted by adipose tissue NK cells thereby indirectly confirming the contribution of the NK facet of innate immunity in the progression of NAFLD to NASH [23]. It was also noted that in certain studies the BMI>50kg/m2 showed remarkable phenotypic and functional changes in NK cells as compared to studies where the pathology of NASH was evaluated in individuals with a median BMI of 35kg/m2. The overall pathophysiologic progression of NAFLD to NASH continuum is summarized in Figure 2.

Limitations

The studies performed have a few limitations. The study participants differ from each other based on geography, comorbid conditions, and lifestyle choices so we cannot apply results to the entire population. The evaluation techniques are also different in each study. A liver biopsy is a touchstone through an invasive procedure and cannot be performed on a large cohort and healthy population for comparison. Due to the associated cost, small groups were studied and not all biomarkers on the inflammatory panel can be tested simultaneously. Also, a few findings can be better evaluated on certain tissue and cell yield. Regardless, we have gathered meaningful information to proceed and explore more preventive and treatment options.

## Conclusions

The systematic review was designed to present the holistic presentation to learn about the basic inflammatory pathways involved in the development of NAFLD and its progression to NASH. We discussed the natural immunity and provocative components to present the current research being done. We would expect these studies to be conducted at a larger scale at the level of all age groups as the epidemic of obesity is affecting all equally and NAFLD and NASH are becoming the most common cause of hepatic morbidity. This enhanced analysis will benefit future researchers and clinicians to focus on the agenda to develop new non-invasive biomarkers for timely diagnosis and treatment orientation to obtain considerable results for improved and new analytical tests and treatment modalities.
